# Ultrastable gold substrates: Properties of a support for high-resolution electron cryomicroscopy of biological specimens

**DOI:** 10.1016/j.jsb.2015.11.006

**Published:** 2016-01

**Authors:** Christopher J. Russo, Lori A. Passmore

**Affiliations:** MRC Laboratory of Molecular Biology, Francis Crick Avenue, Cambridge CB2 0QH, UK

**Keywords:** Electron cryomicroscopy, Single-particle reconstruction, Electron tomography, Gold supports, Cryo-EM, Protein structure

## Abstract

Electron cryomicroscopy (cryo-EM) allows structure determination of a wide range of biological molecules and specimens. All-gold supports improve cryo-EM images by reducing radiation-induced motion and image blurring. Here we compare the mechanical and electrical properties of all-gold supports to amorphous carbon foils. Gold supports are more conductive, and have suspended foils that are not compressed by differential contraction when cooled to liquid nitrogen temperatures. These measurements show how the choice of support material and geometry can reduce specimen movement by more than an order of magnitude during low-dose imaging. We provide methods for fabrication of all-gold supports and preparation of vitrified specimens. We also analyse illumination geometry for optimal collection of high resolution, low-dose data. Together, the support structures and methods herein can improve the resolution and quality of images from any electron cryomicroscope.

## 1 Introduction

Structure determination of biological molecules using cryo-EM has undergone a revolution recently, such that near-atomic resolutions are now possible (Kühlbrandt, 2014). Improved, more stable electron microscopes and new computational methods have contributed to these advances (Henderson, 2015a). But a dramatic improvement has been afforded by the increased quantum efficiency of new high-speed direct electron detectors, resulting in improved images (Cheng, 2015, Henderson, 2015a). It has long been known that cryo-specimens move when irradiated with the electron beam (Henderson and Glaeser, 1985, Dubochet et al., 1988). This results in image blurring and loss of high-resolution information. Direct electron detectors permit tracking of specimen movement during irradiation, allowing realignment of movie frames to reduce (but not eliminate) image blurring (Brilot et al., 2012, Bai et al., 2013, Li et al., 2013). Importantly, these detectors also allow investigation of the origin of specimen movement and the development of new methods to reduce it (Russo and Passmore, 2014a).

**Fig. 1:**
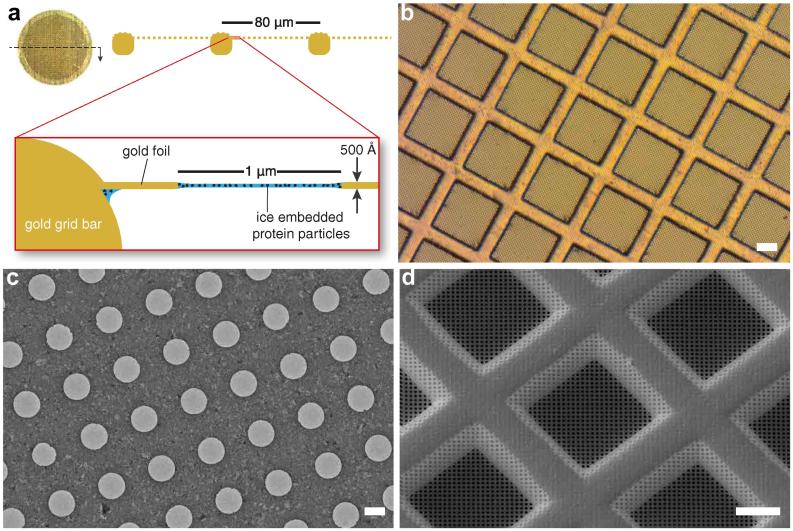
Ultrastable support design. (a) Ultrastable supports, made entirely of gold, comprise a 3 mm disc with a square mesh that supports a ≃500 Å thick foil containing a regular array of micrometer-sized holes. An aqueous protein solution is suspended across the holes and plunge frozen at ≃80 K. (b) Optical micrograph of an all-gold support, showing the square mesh and perforated foil. Scale bar is 25 μm. (c) Transmission electron micrograph taken with 300 keV electrons, showing the perforated polycrystalline gold foil. The holes are 1.2 μm in diameter. Scale bar is 1 μm. (d) Scanning electron micrograph of an ultrastable support taken at 30 degrees tilt from the horizontal axis. Scale bar is 25 μm. Diagram in (a) is reproduced from Russo and Passmore (2014b).

There have been several recent attempts to design supports that reduce the movement of the specimen during irradiation, by changing either the geometry or the material composition of the suspended foil (Vonck, 2000, Rhinow and Kühlbrandt, 2008, Yoshioka et al., 2010, Glaeser et al., 2011, Russo and Passmore, 2014a). We recently showed that much of the particle motion in cryo-EM is due to movement of the support: upon irradiation, supports with a perforated carbon foil over a metal mesh grid move by a surprising amount: 200–400 Å in the direction parallel to the electron beam (Russo and Passmore, 2014b). By carefully analysing the origins of this we designed a support intended to nearly eliminate it, and improve many of the practical aspects of specimen preparation for electron cryomicroscopy. The design (Fig. 1) comprises a circular disk of gold, 3 mm in diameter, having a mesh pattern on which is suspended a thin, polycrystalline gold foil with a regular array of micrometer-sized holes. The design is essentially the same as standard Quantifoils (Ermantraut et al., 1998) except the mesh grid and thin foil are entirely made from gold.

Building upon our previous work (Russo and Passmore, 2014b), here we explain the physical origins of the vast reduction in movement on all-gold supports. We further provide: (1) a practical framework for manufacturing these supports in the laboratory (2) procedures for using them to prepare vitreous specimens and (3) carefully tested methods for collecting optimal data using low-dose techniques specifically optimised for these ultrastable supports. Together these comprise a way to reduce the movement of vitrified biological specimens to less than two ångströms in a typical low-dose micrograph, and thus directly improve the images from any high-resolution electron cryomicroscope.

## 2 Results

### 2.1 Support fabrication

Although we explored different methods to produce an all-gold support, the simplest and most reproducible was to start with a standard carbon foil suspended on a gold grid such as a Quantifoil, C-flat or lab-made perforated carbon support (Ermantraut et al., 1998, Quispe et al., 2007, Adrian et al., 1984). Gold is deposited on the carbon foil by vacuum evaporation, and the support carbon is removed using a low-energy argon–oxygen plasma. A detailed protocol describing the process is provided in Supplementary file 1. After fabrication, the support has the geometry shown in Fig. 1 and has a mass of 0.99 ± 0.03 mg. This means that although the grid is made of a precious metal, the raw material cost is negligible compared to the cost of manufacture or the cost of microscope time and other consumables used in the process.

#### 2.1.1 Mechanical stability

We previously showed that on widely used Quantifoil supports, much of the specimen movement induced by an electron beam is due to motion of the support itself (Russo and Passmore, 2014b). Compared to amorphous carbon foils on gold grids of the same geometry, all-gold supports have as much as a 50-fold reduction in movement parallel to the beam. This results in a twofold reduction in the movement of particles perpendicular to the beam, which is in the plane of the image. But why does gold move less than carbon? One might expect that the gold moves less than carbon because it is thicker and therefore stronger. Previously, we measured the strength of the gold and carbon foils by atomic force microscopy (AFM) and found that exactly the opposite is true; the gold is actually 40 times less rigid at room temperature (10.5 N/cm vs. 412 N/cm) (Russo and Passmore, 2014b). Since the difference in modulus between these two materials will not change by orders of magnitude with cooling (Haynes, 2015), this cannot explain the reduction in movement on the gold foils. To understand this perplexing difference further, we performed a series of experiments on carbon foils to try to pinpoint the origin of the movement.

**Fig. 2:**
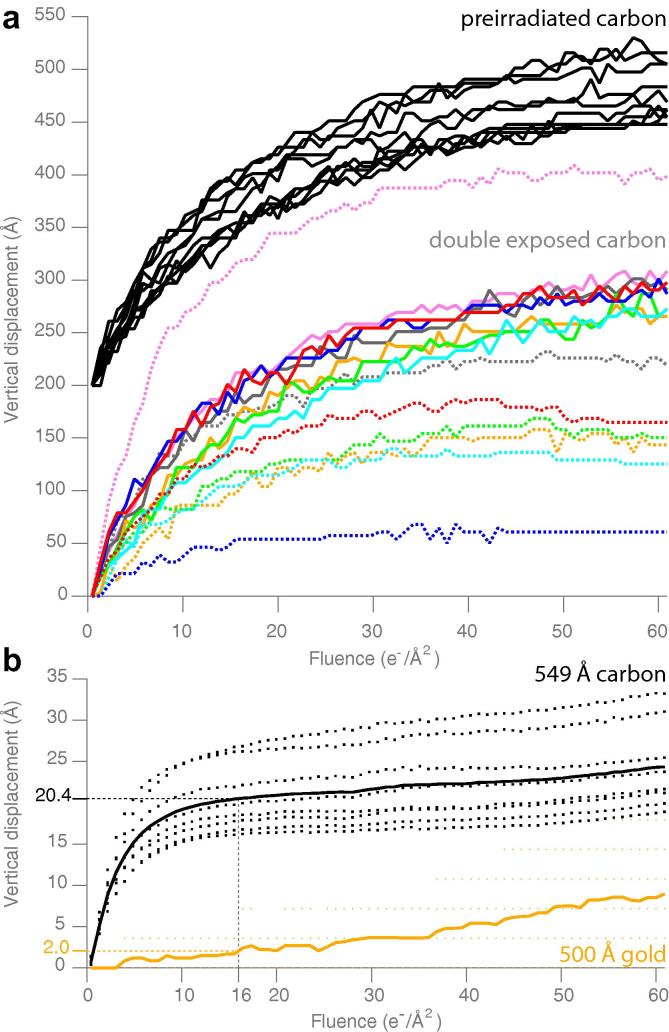
Vertical motion of various support foils irradiated in cryogenic conditions. Pre-irradiation and double exposure (a): black curves are the vertical motion (perpendicular to the plane of the foil) of multiple holes in amorphous carbon after pre-irradiation with 100 $e^{-}/{Å}^{2}$ at 300 keV. Curves are vertically offset by 200 Å for clarity. Coloured curves are the movement of individual holes irradiated twice, where each colour is a single hole and the dotted line is the first exposure and the solid line is the second. Between exposures, the grid was removed from the microscope, warmed to room temperature, re-cooled to 77 K and reinserted in the microscope. Thick carbon foils (b): the vertical motion of a thick carbon foil (∼4× thicker than standard Quantifoils, 549 Å measured by AFM) is shown in (b), black curve. Dotted curves are the individual measurements for 8 different holes, and the solid line is the RMS displacement for the set. Compare to a 500 Å thick gold foil (reproduced from Russo and Passmore, 2014b). All data collected with 300 keV electrons with the support at 80 K.

It has long been known that amorphous carbon, as manufactured, undergoes chemical and physical changes when it is first irradiated by high energy electrons (Miyazawa et al., 1999). Based on this observation, some pre-irradiate the carbon before use with a dose that is large enough to saturate any of the radiation-induced chemical and physical changes in the specimen (∼100 $e^{-}/{Å}^{2}$ at 100 keV (Miyazawa et al., 1999)). This is thought to strengthen, clean and improve the conductivity of the carbon films. Our previous measurements of the vertical motion of carbon foils also show a saturation behaviour to the movement, such that after about 50 $e^{-}/{Å}^{2}$ it nearly stops (Russo and Passmore, 2014b). We tested the effect of pre-irradiation on the vertical motion of the carbon foils in cryogenic conditions, and the results are shown in Fig. 2a. Pre-irradiation at 300 keV using 100 $e^{-}/{Å}^{2}$ makes the trajectories of vertical movement more uniform across the foil, indicating that it does have an effect on either the generation of force by the electron beam or the mechanical response of the foil to this force. Still, it fails to reduce the movement and therefore pre-irradiation is not sufficient to eliminate the motion of the carbon foil.

To further explore the effects of pre-irradiation on the carbon films, we repeated the experiment in a slightly different way: irradiate the carbon around specific individual holes, remove the grid from the microscope, warm it to room temperature, cool it again to 77 K and re-image the same holes. The resulting vertical motion trajectories are shown in Fig. 2a. Again, irradiation makes the holes move more uniformly in the vertical direction, but fails to eliminate the movement. This experiment illustrates the high degree of variability across a grid. Interestingly, there is little correlation in the magnitude of the first and second trajectories, the explanation for which we will discuss below.

Another study showed that increasing the thickness of the carbon foil from the typical 100–200 Å to 350 Å could reduce the movement of 2D crystals of paraffin (Glaeser et al., 2011). The flexural rigidity *D* of a suspended thin film in tension is described by

(1) $$D=\frac{{\mathrm{Eh}}^{3}}{12(1-\sigma^{2})}$$

where *E* is Young’s modulus, *h* is the thickness of the foil, and σ is Poisson’s ratio (Landau and Lifshitz, 1986). Eq. (1) entails that (ignoring the perforations) the flexural rigidity of a thin suspended foil should scale as thickness cubed. So one might also think that simply making the carbon much thicker, as was done previously for paraffin crystals (Glaeser et al., 2011), would be enough to achieve the same results we see on gold. To test this, we repeated the vertical motion tracking experiment for much thicker carbon (549 Å thick), and found that the motion was reduced (22 Å RMS), but it was still about 10 times worse than a 500 Å thick gold foil (2 Å RMS) (Russo and Passmore, 2014b). These data suggest that thick carbon foils might be advantageous over the standard carbon foils, but are less stable than gold foils and still suffer from other shortcomings in the context of cryo-EM (irreproducibility, poor conductivity, instability with time etc.).

#### 2.1.2 Flatness

Together, these experiments led us to conclude that the tension of the foil is more important than the flexural rigidity of the material from which it is made. The reasons for this are at least partially explained by differential contraction of the materials of the support structure during cooling (Glaeser, 1992, Booy and Pawley, 1993, Glaeser et al., 2011). Using the notation of Landau and Lifshitz (1986) and ignoring terms of order greater than one, the relative change in volume caused by deformation during cooling of an isotropic material is

(2) $$u=\alpha (T-T_{0})$$

where $T_{0}$ is the initial temperature, *T* is the final temperature, and α is the thermal expansion coefficient of the material. Using Eq. (2) and tabulated values for α (Haynes, 2015), the relative contractions of gold or copper and amorphous carbon when cooled from 300 to 77 K are 0.3% and 0.1% respectively. Since there is no difference in thermal contraction between the foil and the grid for the all-gold support, the crinkling that was previously observed (Booy and Pawley, 1993) for carbon films on copper grids is eliminated. This means that the gold foil should be flat across the square, and remain so as it is irradiated.

In our previous paper on ultrastable supports, we proposed a simple model for the 2-fold reduction in horizontal movement of the particles based on the 50-fold reduction in vertical movement of the support layer (Figure S4 in Russo and Passmore, 2014b). The model is based on the bending of the crinkled foil and ice during irradiation.

**Fig. 3:**
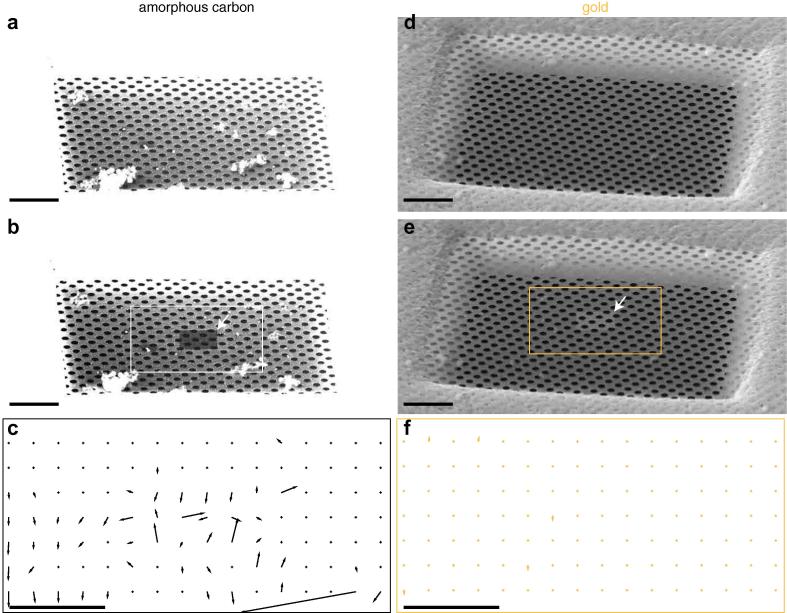
Movement of gold vs. carbon foils at cryogenic temperatures. Scanning electron micrographs of amorphous carbon ((a) & (b)) and gold ((d) & (e)) foils suspended on gold grids. Micrographs (b) & (e) are of the same region as (a) & (d) after high-dose irradiation (∼1000 $e^{-}/{Å}^{2}$) of a local region of the foil indicated by the arrows. Comparison of the before and after micrographs (Supplemental videos 1–4) show clear movement and bending of the amorphous carbon foil after high-dose irradiation, while the small amount of movement in the gold foil is due to thermal drift of the stage. These movements are quantified in vector plots in (c) & (f); movement vectors were determined for 128 × 128 pixel regions of the micrographs and their magnitude is magnified 4× in the plot. Micrographs were taken using 30 keV electrons and an off-axis Everhart–Thornley secondary electron detector biased to +250 V, with the specimen cooled to 98 K. Tilt of the grid is 59° to the horizontal, with equal magnification and beam current in all micrographs. The brightness and contrast was adjusted to make the carbon foil visible; videos have the unmodified version. Scale bars are 10 μm.

To confirm this explanation, we imaged gold and carbon foils under identical cryo-conditions and with identical geometries, using a scanning electron cryomicroscope (Fig. 3 and Supplemental videos 1–4). By mounting the supports at a 59° angle to the beam, it was possible to directly visualise the bends and contours of the suspended foil. After irradiating a small region of the foil with a large dose of electrons (∼1000 $e^{-}/{Å}^{2}$), the gold foil remained unchanged, but the carbon foil flexed and bent. It did so in a non-uniform way, explaining the variability in the trajectories of Fig. 2. This confirms the notion that at least part of the motion reduction on gold foils is due to reduced mechanical deformation during irradiation with the electron beam.

#### 2.1.3 Electrical resistivity

**Fig. 4:**
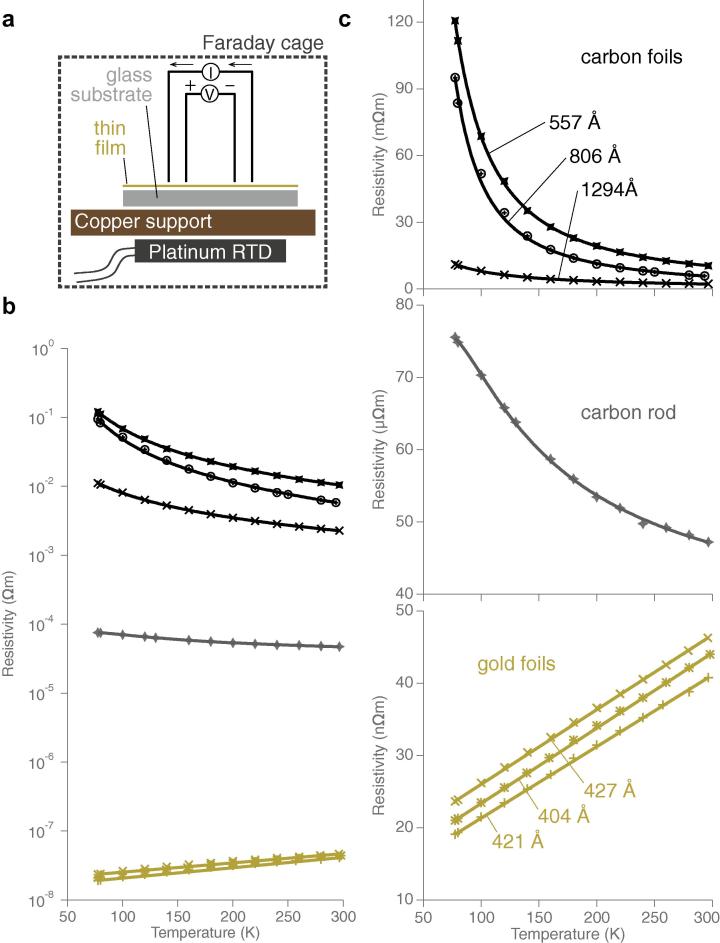
Resistivity of thin foils of polycrystalline gold and amorphous carbon from 298 K to 77 K. A schematic diagram of the cryo-four-point probe instrument (a) used to measure the electrical resistance of thin films versus temperature. It consists of four gold spring pins that make contact to the thin film which is supported on a sample stage made of gold-plated copper that is attached to a calibrated platinum temperature sensor (RTD). The entire apparatus is enclosed in a gold-plated copper cylinder that acts as a Faraday cage, to reduce noise, and a cryostat to maintain a uniform temperature around the probes. Electrical feedthroughs attach the probes and temperature sensor to calibrated source meters. Panel (b) shows measurements of the conductivity vs. temperature of three separate evaporations of amorphous carbon (upper black curves), a 3 mm carbon rod (middle grey curve) and three evaporated gold foils (lower gold curves). The points are the average resistivity at a particular temperature and the curves are fits to the data. Each measurement was made 10–12 times and the error on both axes (S.E.M) was smaller than the size of the markers on the plot. Both the temperature and voltage/current were calibrated against traceable sources to be accurate to <1 part in $10^{5}$. Plots in (c) are the same as in (b) but magnified and rendered on a linear scale to show the form of the fits. The thickness of each foil, measured by AFM as described previously (Russo and Passmore, 2014a), is indicated.

Since the buildup of charge is a likely origin of the force on the suspended foil and may cause distortion of the beam during imaging, we wished to accurately characterise the electrical properties of the support foils under the conditions in which they are irradiated. Previously it was shown that the typical thin films of amorphous carbon used as EM support layers are semiconducting (Rhinow and Kühlbrandt, 2008, Larson et al., 2011), and can have widely varying conductivity at room temperature (Henderson, 2015b, Larson et al., 2011). Inspired by these experiments, we built a cryo-four-point probe instrument to accurately measure the resistivity of thin films. The instrument is diagrammed in Fig. 4a; it comprises four gold spring pins in a linear array that make contact with the film, held on a copper sample stage. The stage is enclosed in a copper chamber that is contiguous with the shielding of the electronic circuits to remove any external electric fields and sources of noise. A calibrated platinum temperature sensor was attached to the copper sample stage to accurately measure the temperature of the sample during resistivity tests. The entire apparatus was designed to be cooled to liquid nitrogen temperatures, allowing measurement from 300 to 77 K. This, combined with contact-mode AFM to measure the thickness of the foil, allowed precise measurement of film resistivity vs. temperature.

We used the four point probe to measure the sheet resistance, *R* of carbon and gold foils between 300 and 77 K (Fig. 4b and c). The foils were subsequently cleaved with adhesive tape, and their thickness, *h* was measured by AFM. The resistivity, ρ, was then calculated using

(3) $$\rho =\mathrm{hR}=h\frac{V}{I}C$$

where *I* is the applied current, *V* is the measured voltage and the geometric factor, *C*, was taken from tabulated values for the probe geometry (Sze, 1981).

The measurements of resistivity vs. temperature for three different carbon foils, prepared in separate evaporations using the same conditions, are shown in Fig. 4b and c. The thin films of amorphous carbon have a high resistivity which can change by orders of magnitude from one evaporation to the next. Furthermore, the resistivity of the carbon increases exponentially when cooled to 77 K. This exponential behaviour indicates that the amorphous carbon is a semiconductor whose conductivity depends on carrier concentration rather than mobility (Sze, 1981). From the fits, the resistivity was in the range 0.1–0.01 Ωm for films prepared identically with evaporation base pressures in the $10^{-7}$ torr range. This indicates that during typical evaporations used for making carbon support foils, the number of impurities incorporated within the foil during evaporation, and also the carbon–carbon bonding structures can vary widely, as has been previously observed in other studies of condensed carbon materials (Franklin, 1951, Blue and Danielson, 1957, Gjsonnes, 1960). The resistivity of a 3 mm carbon rod, of the sort we use to create these carbon films is lower than any of the thin films, probably because it is of higher purity and surface effects are eliminated in this geometry. This provides an estimate of the lower limit of thin carbon film resistivity. We find it is of the order $10^{-4}$ Ωm, and behaves as a semiconductor–the resistivity increases as the temperature falls to the cryogenic regime.

In contrast, the resistivity of the gold foils used to make ultrastable supports was less than 50 nΩm, and decreased by a factor of two when cooled to liquid nitrogen temperatures. We note that this is orders of magnitude less than amorphous carbon films and the previously proposed films made from titanium silicide (1 μΩm at 77 K, Rhinow and Kühlbrandt, 2008) or silicon carbide (300 μΩm at 77 K, Yoshioka et al., 2010).

### 2.2 Structure of the metal foil

The most important factors governing both the conductivity and mechanical stability of thin films of gold are the purity of the metal and the size and distribution of individual crystal grains in the polycrystalline foil (Wise, 1964). The purity of gold is easily controlled using high quality source material (>99.95% purity), and low residual vapour pressure during deposition ($∼10^{-6}$ torr). Since gold resists oxidation or chemical reaction with the typical residual gases in a vacuum chamber (nitrogen, water, hydrocarbons, noble gases, etc.) it is relatively easy to obtain very high purity films using only modest vacuum chambers ($10^{-7}$ torr range) with simple tungsten filament thermal evaporation sources like the one shown in Supplementary file 1. This is in contrast to many other metals like tungsten, molybdenum, platinum and titanium, which require higher temperatures, more sophisticated sources, or better vacuum to achieve reproducible films with high purity and uniformity.

**Fig. 5:**
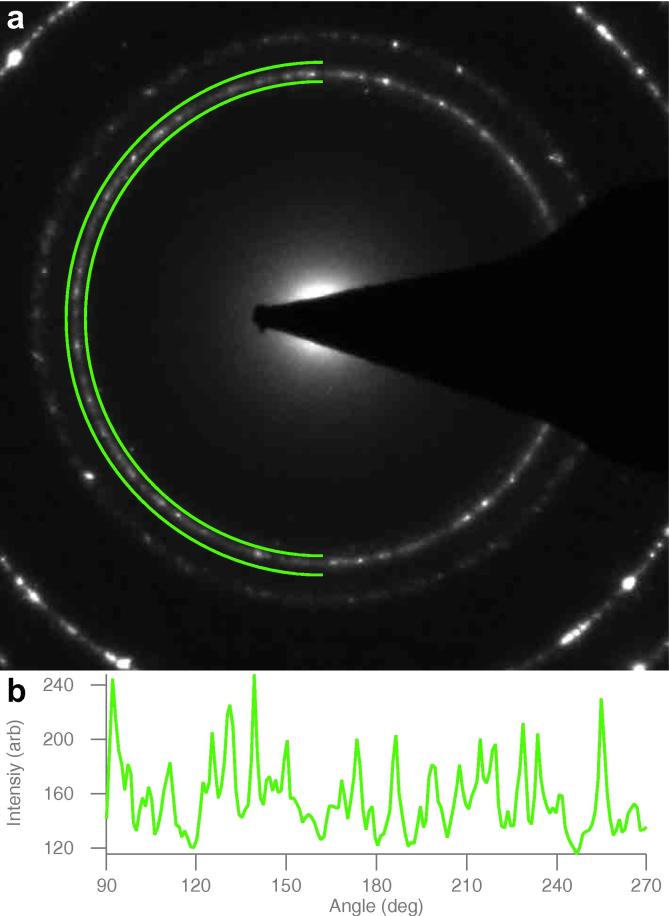
Polycrystalline grain structure. The average number of grains per unit area in the suspended gold foil was estimated using selected area electron diffraction (a) of an ∼1 μm^2^ area between the holes. To determine the number of grains, the number of reflections in a semi-annulus containing the 111 ring (green region) was counted from the number of peaks (b), and this taken as the approximate number of crystal grains within the region.

The size of the grains in the thin film of metal is determined by the rate of deposition and the temperature of the substrate during evaporation (Sree Harsha, 2006). This is because the vaporised gold atoms strike the carbon template layer with significant energy (of order $k_{b}T$, which is 0.12 eV for gold at its melting point), and so are able to diffuse around on the surface of the film some distance before they cool enough to bond to another atom of gold. Increasing the deposition rate will reduce the time which each atom has to diffuse, thus increasing the number of grains and decreasing their size. But increasing the rate also increases the temperature of both the vaporised atoms and the substrate due to inferred radiation from the source; this will in turn increase the diffusion of the atoms on the surface and increase the grain size. In the evaporation chamber we used for fabrication, there is no active cooling of the substrate; by carefully controlling the source to sample distance and the deposition rate using a crystal thickness monitor, we were able to empirically test a range of conditions, and thus achieve a highly reproducible grain structure within the foils. To monitor the variation in grain size from one batch of foils to the next, we designed an assay using selected area electron diffraction. By counting the number of grains per unit area (Fig. 5), we could quickly ensure that the properties of the foil were consistent from batch to batch. Using the procedure detailed in Supplementary file 1 to make a 400–500 Å thick foil, the number of grains per unit area that diffracted was 29 ± 4 grains/μm^2^ (s.d. for 3 batches). Note that this is really a lower limit, as some grains will not diffract because they are too small or not aligned close enough to a zone axis to contribute a peak.

### 2.3 Specimen preparation

**Fig. 6:**
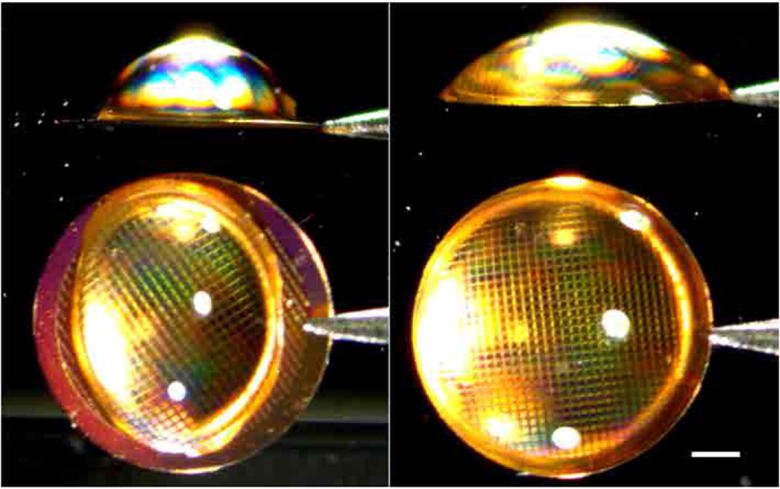
Low-energy plasma treatment to decrease hydrophobicity. Image shows a 3.0 μl droplet of pure water on the surface of an all-gold support as manufactured (left) and immediately after a 20 s argon:oxygen plasma treatment (right). Micrographs are taken using a first surface mirror at an angle of 45 degrees placed adjacent to the grid so both the top and side views are imaged simultaneously. Scale bar is 0.5 mm.

Unlike other metals, gold is biocompatible, inert, and can be easily tailored to control the adsorption of proteins or cells (Mrksich et al., 1996). To prepare vitreous specimens, a few microliters of protein solution are applied to the support, excess solution is removed by blotting, and the specimen is then rapidly cooled by plunging into liquid ethane. To obtain a sufficiently thin and uniform layer of vitreous ice, the support needs to be hydrophilic. Typically this is achieved by exposing it to a low-energy plasma made from air or a specific mixture of gases like argon and oxygen. Like carbon supports, gold is not particularly hydrophilic, but can be easily made more so using standard plasma treatment protocols. Fig. 6 shows a 3 μL droplet of water applied to an all-gold support before and after a 20 s plasma treatment using a 9:1 argon:oxygen mixture as detailed in the methods. Using this setup, we measured the air–water–gold contact angle for a 1 μL droplet on an all-gold grid before and after plasma treatment. The contact angle was reduced from 82 ± 8° to 27 ± 6° (s.d., n=6). The decrease in contact angle demonstrates a reduction in the gold–water surface energy and improved wetting after plasma treatment. In addition, gold is easily modified using standard self-assembled monolayers and other surface modification protocols to control, and thus prevent, binding of biological specimens to the surface (Prime and Whitesides, 1991, Ostuni et al., 2001). Modified gold surfaces have been used in cryo-EM to improve the distribution of proteins within the holes (Meyerson et al., 2014).

Often, an additional layer of thin amorphous carbon is placed on top of a perforated carbon foil to improve the distribution and orientation of the particles within the holes. This is also possible with the all-gold supports described here and we include a procedure for float-transferring thin films of amorphous carbon in Supplementary file 2. In practice, we have found that adding 20–30 Å thick layers of carbon on the gold foils does not degrade the performance of the supports in reducing motion. This is not surprising when one considers that typical thin films of ice are 100–1000 Å thick; adding 20 Å of carbon does not have a large effect on the specimen movement as it is dominated by the movement of the ice.

Finally, we note that improper handling can damage any grid, particularly as it can stretch and distort the grid and compromise the tension of the suspended foil within an individual square. All-gold supports are in practice, less fragile than Quantifoils. Although damage during handling should be avoided with any specimen support, the deleterious effects of compromised structural integrity are even more important for all-gold supports because of their substantially reduced motion.

### 2.4 Data collection

Once one has prepared and mounted the specimen in the microscope cryostage without damage, what is the optimal way to collect data? While the answer to this question is bound to evolve with further improvements in the microscope, detector and specimen support, we can offer some guidance based on a series of measurements of particle movement under different conditions.

#### 2.4.1 Focusing

During low-dose imaging, focusing the objective lens on the plane of the specimen is usually done in advance using an adjacent region of the support foil to minimise the exposure of the region of interest (Williams and Fisher, 1970). The same technique is used for the all-gold supports, but there are two differences with respect to carbon: (1) Gold is a polycrystalline metal and therefore does not exhibit Thon rings (Thon, 1966). (2) The gold foils are flatter than typical carbon so focusing can be done less often.

**Fig. 7:**
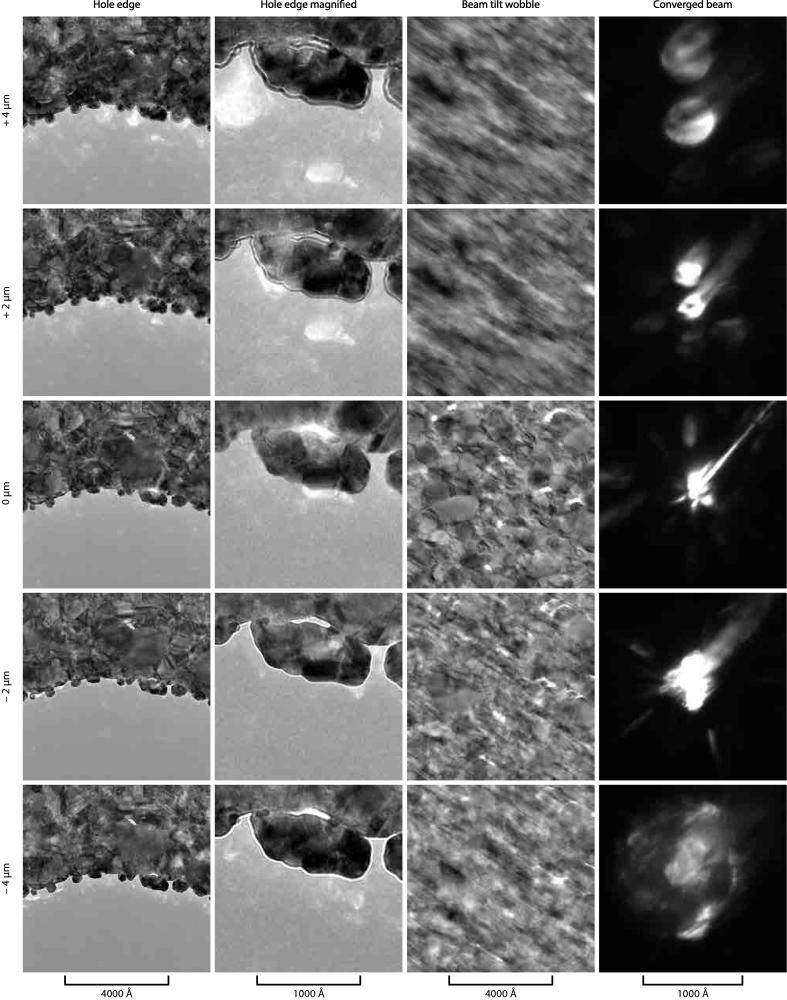
Determining focus using a gold foil. Defocus tableaus at 300 keV of micrographs showing various ways to determine the position of the suspended foil relative to the focal plane of the objective lens. These include the delocalised lattice fringes from the gold crystals at the edge of the hole (bright patches, left two columns), Fresnel fringes at the edge of the hole (apparent in the left middle column), the image shift during beam tilt wobble (middle right column) and the convergence of the diffracted beams with the incident beam converged on the specimen (right column). The objective aperture can be used to exclude all but the first lattice reflection to make the bright patches easy to identify. Scale bars are indicated at the bottom of each column and nominal defocus to the left of each row.

There are several distinct ways to determine the focus of a polycrystalline film of metal. Four are shown in Fig. 7, and any of them can be used in the context of low-dose imaging. (1) The beam tilt can be oscillated about 0 deg; then the plane for which the image shift is minimised (Fig. 7, column 3) is the in-focus condition ($C_{1}=0$) (Reimer and Kohl, 2008). (2) Each individual crystal of gold in the foil diffracts electrons away from the unscattered beam. These diffracted electrons are delocalised from the primary image of the crystal along an angle $\theta_{B}$ according to the Bragg equation

(4) $$2d\sin\theta_{B}=\lambda$$

where λ is the wavelength of the electron and *d* is the lattice spacing. Since gold has a face centred cubic structure with a lattice constant of 4.08 Å, the smallest plane spacing, 111, is for d=2.35 Å (Haynes, 2015). For 300 keV electrons, the wavelength is 1.97 pm, which is much less than *d*, so we can use the small angle approximation for $\sin\theta_{B}$. Then Eq. (4) entails that this reflected beam of electrons, which appears as a bright spot in a phase contrast image, is delocalised a distance *r* from the crystal according to

(5) $$r≃\frac{\lambda C_{1}}{2d}$$

where $C_{1}$ is the defocus. In practice, the “bright spots” from the diffracted beams are easy to see even on a phosphor screen with a low-resolution microscope (Fig. 7, columns 1 & 2). This makes it fast and easy to bring the specimen to a desired value of defocus with sub-micron accuracy even without the use of digital cameras or real-time fast Fourier transforms. Note that if an objective aperture is used this reflection may be excluded depending on the maximum angle it subtends; in practice we tend to use an objective aperture that admits frequencies that include at least the first ring of reflections so they can be used for focusing. We have found no obvious difference in the radiation-induced motion for data collected with and without an objective aperture. (3) A similar diffraction effect occurs when the beam is brought into convergence on the specimen (Fig. 7, column 4); the resulting diffraction pattern converges to a central spot as the specimen is brought into focus. (4) The electron Ronchigram from the converged beam is also very sensitive to the defocus (Reimer and Kohl, 2008) and can be used to determine focus plane, but this is less practical in typical bright field phase contrast microscope setups. (5) The Fresnel fringes at the edge of a hole in the foil are easy to see and can also be used to determine the magnitude and sign of the defocus coefficient (e.g. Fig. 7, column 2) (Reimer and Kohl, 2008). (6) Another simple way to focus the lens on the specimen is to use the Thon rings from a specimen hole that contains protein. One can also use the Thon rings from the amorphous ice itself (McMullan et al., 2015). In practice, for manual data collection we use the delocalised lattice fringes (2) and for automated data collection we use beam tilt wobble/image shift (1), both of which are easier and faster on gold than on typical amorphous carbon foils. Final values of defocus used for reconstruction are determined from the micrographs using standard CTF fitting algorithms (Mindell and Grigorieff, 2003).

We measured the variation in defocus across a typical grid square during an automated data collection run over an area of 30 μm × 30 μm and found it was ±0.6 μm (s.d.) from the mean value. With this amount of variation, it is unnecessary to determine focus for each hole; once every 10–20 μm is more than sufficient and the residual variation can be used to cover the zeros in the contrast transfer function for a particular dataset. This improves the efficiency of the electron microscope for both manual and automated data collection as less time is spent bringing the specimen into focus.

#### 2.4.2 Illumination geometry

**Fig. 8:**
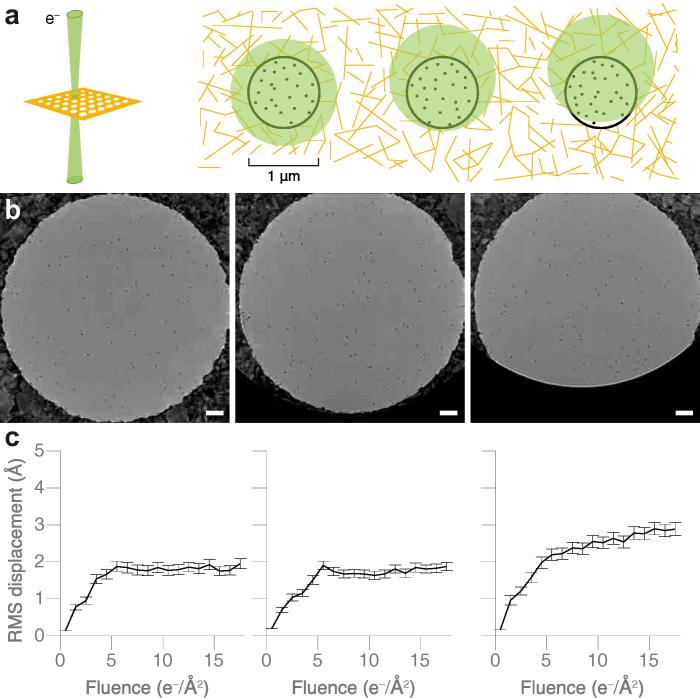
Effect of electron beam illumination geometry on the movement of particles in vitreous ice. Panel (a) shows the geometries tested where an individual grid square is depicted on the left, with the transmitted electron beam shown in green. A diagram of the polycrystalline gold foil (gold lines) supporting a thin film of vitreous ice within the holes (circles) containing individual particles (black dots) is shown on the right. The three different electron illumination geometries are shown in green. (b) Electron micrographs for each beam illumination geometry, each showing a hole in the gold foil containing 10 nm gold fiducial particles suspended in vitreous ice. Images were taken with typical illumination conditions (300 keV, 16 $e^{-}/{Å}^{2}$/s and 80 K). Scale bars are 100 nm. Panel (c) shows the RMS particle displacement corresponding to each geometry.

Low-dose imaging is designed to minimise specimen exposure before the actual imaging data is collected on the region of interest (Williams and Fisher, 1970). But motion and charge accumulation blur the image before radiation damage destroys the specimen (Henderson and Glaeser, 1985, Dubochet et al., 1988, Brilot et al., 2012, Russo and Passmore, 2014b). It has long been known that the shape, position and intensity of the electron beam during low-dose imaging can affect the quality of the micrograph (Henderson and Glaeser, 1985, Downing and Glaeser, 1986, Bullough and Henderson, 1987, Avila-Sakar and Chiu, 1996). Unwin and colleagues proposed that the optimal geometry for illuminating the specimen during low dose imaging was a cylindrically symmetric beam that is centred on the hole and encompasses an annulus of the support foil (Miyazawa et al., 1999). The idea is simple: by irradiating the specimen hole symmetrically, any of the complicated forces and fields that lead to image blurring will also be symmetric and therefore reduced. During initial work with all-gold supports, we tested many different illumination geometries and immediately noticed that the best data was from this symmetric illumination condition. To further explore this we conducted particle tracking measurements under different illumination conditions (Fig. 8). Nanogold particles were irradiated with a cylindrical beam of electrons that was centred: (1) on the centre of the specimen hole (2) slightly offset from the centre of the hole or (3) largely offset from the centre of the hole, such that the entire suspended ice region is no longer irradiated. The result of the tracking experiments is shown in Fig. 8c. As soon as the beam no longer covers the entire ice region, the particles in the image begin to move more and the image quality degrades significantly. We therefore recommend symmetrically illuminating a region encompassing the entire hole and an annulus of the gold foil around the hole. We speculate that this reduces the differential buildup of charge on the suspended ice, thus reducing any semi-static lensing effects on the beam (Curtis and Ferrier, 1969). Illuminating the region of gold directly adjacent to the ice may also improve the images through the generation of low-energy secondary electrons that can neutralise the buildup of positive charge on the ice. Secondary electrons have been used previously to reduce image blurring (Berriman and Rosenthal, 2012) and we expect a similar effect is present when the gold near the suspended ice is illuminated, particularly as gold has a relatively high secondary electron yield relative to both carbon and ice (which is apparent in the scanning electron micrographs of Fig. 3 and Supplemental videos 1 & 2) (Reimer and Kohl, 2008). These secondary electrons are created adjacent to the region that is imaged so are likely to be recaptured into the positively charged ice layer.

## 3 Discussion

Although it has long been known that radiation-induced specimen movement can severely impact the quality of electron cryomicrographs, it was only recently appreciated what role the specimen support had in this degradation. Typical Quantifoil supports move a large amount upon irradiation, and this is the source of much of the specimen movement.

Crinkling of carbon foils due to differential contraction of the metal grid and the carbon film explains why the amount of movement varies from one hole to the next and has no strong correlation with either the location on the grid or the position within a square. This also explains why re-irradiating the carbon around the same hole after warming up the grid and re-cooling still leads to motion of the foil during irradiation which is inconsistent, even for the same hole in the same position on a specific grid. Amorphous carbon films are semiconducting and have widely varying resistivity as manufactured. This resistivity increases exponentially as the carbon is cooled to liquid nitrogen temperatures. Accumulation of static and semi-mobile charge on this poorly conducting, thin material may also have a role in the instability and image blurring on carbon supports. Further work will be required to quantify this effect and ascertain the degree to which charge accumulation causes blurring.

Making the support entirely from gold improves image quality in at least three ways: (1) The differential contraction of different materials (e.g. carbon on copper) during cooling is eliminated. (2) The foil is highly conductive at all temperatures of interest and does not accumulate static or semi-mobile charge that can potentially distort the images. (3) Irradiating the gold adjacent to the suspended ice generates secondary electrons close to the suspended ice which may neutralize accumulated positive charge in the specimen.

In practice, it is easier and more accurate to determine focus and collect data using an all-gold support. In our experience, the ice is more reproducible, the foil is flatter and the high contrast of the gold facilitates grid surveys and automated data collection. The only disadvantage we are aware of compared to carbon is lack of Thon rings from the foil material. These rings are often used to measure and correct high-order anisotropic lens alignments like stigmation; in practice this is not a problem since these optical alignments are best done with a calibration grid prior to imaging the specimen, and on modern microscopes, the alignments are stable from 10’s of hours to days. It is also theoretically possible that the additional secondary electrons generated by irradiating the gold could damage molecules near the surface of the ice; we have not seen any evidence of this in practice.

For a support like the one depicted in Fig. 1, the cross-sectional open area is 0.5 mm^2^. For a typical specimen that has particles every 200 Å, this means the total number of usable particles for a perfect support would be of order $10^{9}$. This more reproducible support will facilitate collection of large amounts of data from a single grid using automated data collection. Still, structural heterogeneity of the specimen and the ability to control the distribution and orientation of the particles within the holes limits structure determination for many molecules. Future work will focus on incorporating tuneable, conductive monolayers like graphene, particularly as graphene is known to make ohmic contact to gold (Sundaram et al., 2011) and will allow improved control of the distribution of particles within the ice (Russo and Passmore, 2014a).

One might consider materials besides gold to use for the specimen support. Any candidate material should be non-oxidizing, radiation hard, chemically inert and highly conductive even at cryogenic temperatures. This limits one to gold, platinum and palladium. Platinum and palladium supports may be as stable after cryo-plunging, but are more difficult to manufacture and are more likely to catalyse chemical reactions within the specimen. Copper, aluminium or silver will also be highly conductive at liquid nitrogen temperatures, but will likely be more reactive and more likely to accumulate surface charge due to the surface oxide layer present on these metals after exposure to air. Making the grid out of a metal that contracts less than the suspended carbon has been considered previously (Booy and Pawley, 1993), but we suspect it will be difficult to match the thermal expansion coefficients with sufficient accuracy to make this practical. In addition, this will not solve the other problems with amorphous carbon: its poorly reproducible electrical properties, the chemical changes that occur to its structure during irradiation with high energy electrons, and its poor conductivity at liquid nitrogen temperatures.

Many recent cryo-EM studies that have achieved high resolution have relied on discarding large amounts of data: as much as 95% or more of the grids, micrographs or particles are discarded during collection or processing. Excluding specimen heterogeneity and ice thickness variation, we suspect that most, if not all, of this discarded data is due to movement and electrical instabilities in the specimen during irradiation. And given the understanding of the origins of particle movement described here, it is now easier to rationalise why the data quality is so variable from grid to grid, square to square, and hole to hole.

Empirical methods of discarding poor quality data are reasonable and often effective for structure determination, provided care is taken to validate the final density maps. Still, there is an inherent danger in the context of developing new methods; anytime one discards a large portion of the grids, squares, micrographs, or individual particles from a particular dataset, any comparisons based on those data are potentially subject to a sampling bias. Then because of bias introduced by the selection process, one risks erroneously attributing the reason for improved resolution for a map calculated from those data. By improving the specimen support, we do not just increase the resolution and quality of each micrograph and improve the efficiency and throughput of the microscope. We also strengthen the reliability and reproducibility of cryo-EM experiments in general, placing interpretations of structural information on a stable foundation.

## 4 Materials and methods

### 4.1 Fabrication of gold supports

The detailed protocol for manufacturing gold supports is provided as Supplementary file 1. Briefly, we use Quanitfoil R1.2/1.3 Au grids (that have a perforated amorphous carbon foil suspended over gold grids) as templates: We first evaporate gold onto the carbon layer and subsequently remove the carbon using a plasma. Template grids should be carefully inspected using an optical microscope to eliminate any with defects - all should be flat, continuous and without dust or lint. For evaporation, we use a thermal evaporator equipped with a turbo pump and cold trap, with $10^{-7}$ torr base pressure and a thermal point source made from high purity (99.99%) gold wire (shown in Supplementary file 1). Any impurities in the gold or from residual gas in the chamber will affect the quality of the gold foil. The crystal grain size in the gold foil is affected by the deposition rate and substrate temperature so these should be carefully controlled. Deposition rate and gold film thickness can be monitored using a crystal thickness monitor, and the distance from source to sample affects heating of the substrate during evaporation. To calibrate this, and to accurately measure the gold foil we use atomic force microscopy as previously described (Russo and Passmore, 2014a). After evaporation of 450 Å gold, the carbon template layer is removed by low-energy plasma with an argon:oxygen plasma. Gold is evaporated onto the carbon side of the template grids. Thus, even after plasma removal of the template carbon, some carbon will remain trapped between the gold foil and the gold grid bar. It is also possible to evaporate gold onto the bottom side but the thickness of the grid bar makes it difficult to obtain a gold foil that is in contact with the grid bars on all sides, thus requiring a more complicated series of evaporation steps. We found no advantage to supports prepared in this way and recommend the simpler method of evaporating on top of the carbon layer. The entire procedure can be completed in a few hours. Grid mass was measured using a calibrated microbalance (Mettler Toledo XS105); the mean of ten measurements was taken as the average mass and the standard deviation taken as the error. All-gold supports manufactured in this way are also commercially available from Quantifoil Micro Tools GmbH under the name UltrAuFoil.

### 4.2 Four-point probe resistivity measurements

Specimens were prepared by thermal evaporation onto 18 mm square glass coverslips (Zeiss No. 1.5). Each foil was mounted in the probe instrument, the Faraday cage was enclosed around the device and the measurement taken by applying a fixed current to the outer two probes and simultaneously measuring the voltage between the inner two probes using a calibrated source meter (Kiethley 2450). Each measurement for a particular temperature was made 10–12 times, and the mean taken as the actual value. Temperatures were measured using a calibrated, NIST traceable platinum resistor (Lakeshore Cryotronic) attached to the copper substrate stage. The resistance of the temperature sensor was measured in a four-wire configuration using a commercial source meter (Lakeshore Cryotronic 211). The absolute error in the temperature measurement was estimated to be less than ±24 mK in this range of temperatures. To cool the specimen, the Faraday enclosure was purged with dry, cold nitrogen gas and then the entire apparatus was placed in a stainless steel dewar. Liquid nitrogen was slowly added to the dewar, and the resistivity and temperature measurements were recorded as the instrument cooled to the temperature of liquid nitrogen. After warming back to room temperature, the thickness of the foil was measured by first cleaving it with adhesive tape (3 M Scotch Crystal), and then imaging the cleaved edge by contact mode atomic force microscopy (Asylum Research) as described previously (Russo and Passmore, 2014a). (Note: we neglect the thermal contraction during cooling for these resistivity measurements as it will change the thickness by <0.3%). The thermal expansion coefficients (α in Eq. (2)) are 16.5, 14.2 and 4 μm/(mK) for copper, gold and amorphous carbon respectively (Haynes, 2015). Note: reported values for carbon vary from 2–8 so a value of 4 was taken as a reasonable estimate for the differential contraction calculation.

### 4.3 Support micrographs

Optical micrograph in Fig. 1 was taken using epi-illumination through a 10× objective lens (Zeiss EC Epiplan-Apochromat) using a commercial microscope (Zeiss Axiophot) and camera (Zeiss AxioCamERc5s). Scanning electron micrographs were collected using an FEI Scios FIB/SEM instrument using 30 keV electrons, 6144 × 4376 pixels, a 32 μm aperture, a nominal magnification of 5 k× and a dwell time of 5 μs, a working distance of 7 mm; the secondary electrons used to create the image were collected using an Everhart–Thornley detector mounted near the column. For each experiment, the two micrographs were aligned with each other by cross correlation to remove drift, cropped to 80 μm wide and binned 4× to create the image for the figure. Beam current was measured using a Faraday cup mounted on the specimen stage. For the trajectory calculations, after drift correction on the complete micrographs, the selected region of the image stack was subdivided into individual 128 × 128 × 2 pixel stacks, and the movement in each was measured by cross correlation. These were then used to generate the vector plots in the figure. Each vector magnitude was multiplied by four to make them easier to see in the plot. Transmission electron micrograph in Fig. 1 was taken using 300 keV electrons on a Gatan K2 direct electron detector operated in super-resolution counting mode in a FEI Titan Krios.

### 4.4 In-situ contact angle micrographs

Images were taken using a trinocular dissecting microscope (Zeiss Stemi 200-C) attached to a digital camera (Zeiss AxioCam ERc5s). The specimen support was clamped in place using a pair of tweezers (Dumont N5) and placed with the plane of the support parallel to the focal plane and adjacent to a first surface mirror (Thorlabs) that was mounted at 45° to the same. This allowed simultaneous imaging of the support from the side and the top in the same field of view. A 1 or 3 μL droplet of pure water (18 MΩ deionised) was added to the support and then imaged. The grid was then dried with filter paper, treated with a 20 s 9:1 Ar:O_2_ plasma (in a Fischione model 1070, 31.0 SCCM gas flow, 70% power, 1.9 $\times 10^{-2}$ torr, 39.8 W forward power, <2 W reverse), and then the process was repeated to create the second micrograph. We found that once plasma-treated, the change in hydrophobicity lasted on the order of 1 to 2 h when kept in a clean dish in air.

### 4.5 Defocus tableau micrographs

Micrographs of a gold support foil were imaged in a Krios microscope (FEI) with 300 keV electrons and using a Falcon II direct electron detector or a phosphor screen and flu-camera (CBED images). Note there is a slight offset from the nominal 0 of defocus and the actual focal plane.

### 4.6 Vertical motion tracking

Vertical motion of supports was measured as previously described (Russo and Passmore, 2014b). Briefly, the support was cooled to 77 K, mounted in a FEI Titan Krios, tilted to 30°, and irradiated under typical conditions (300 keV, 16 $e^{-}/{Å}^{2}$/s symmetric uniform illumination). Movies of the support were collected and the movement of the edge of the foil perpendicular to the direction of tilt was tracked to determine the amount of vertical motion.

### 4.7 Illumination geometry particle tracking

Particles were tracked as described previously (Russo and Passmore, 2014a), but in this case using protein-A labelled nanogold particles that are typically used as fiducial particles in tomography measurements (Bharat et al., 2015). For the three cases, no offset, small offset and large offset, there were 338, 328 and 313 particles respectively, tracked from three separate movies of three holes for each condition.

## Supplementary data

Supplementary file 1 Experimental protocol for the fabrication of ultrastable gold supports by thermal evaporation.

Supplementary file 2 Experimental protocol for the float transfer of thin films of carbon to ultrastable supports.

Supplementary video 1 Animated before and after scanning electron cryomicrographs of a suspended, perforated amorphous carbon foil irradiated with a high dose in a local region under cryogenic conditions. Field of view is 80 μm wide and tilt of specimen is 59° along the horizontal axis.

Supplementary video 2 Animated before and after scanning electron cryomicrographs of a suspended, perforated gold foil irradiated with a high dose in a local region under cryogenic conditions. Field of view is 80 μm wide and tilt of specimen is 59° along the horizontal axis.

Supplementary video 3 Enlarged view of the local region irradiated in Video 3, showing the bending and movement of the foil. Irradiated patch is 5 μm wide.

Supplementary video 4 Enlarged view of the local region irradiated in Video 4, showing the bending and movement of the foil. Irradiated patch is 5 μm wide.
