# Supplementary material for: Ultrastable gold substrates: Properties of a support for high-resolution electron cryomicroscopy of biological specimens
Source: J Struct Biol. 2016 Jan;193(1):33–44. doi: 10.1016/j.jsb.2015.11.006 (PMC4711342; doi:10.1016/j.jsb.2015.11.006)
Supplement: Supplementary file 1 — Experimental protocol for the fabrication of ultrastable gold supports by thermal evaporation. [file mmc1.pdf]

# Gold evaporation procedure

**Reference:**
**Ultrastable gold substrates: Properties of a support for high-resolution electron cryomicroscopy of biological specimens**
Christopher J. Russo & Lori A. Passmore

## Summary

This procedure describes how to fabricate ultrastable gold supports for electron microscopy using a holey carbon support as a template.

## Warning

This procedure involves using high voltage, high temperatures and equipment under vacuum. It is for use by experienced scientists who know how to handle such equipment and have done all appropriate local safety training and risk assessments, etc. Wear safety glasses and use at your own risk.

## Materials

| Description | Amount | Vendor | Part number |
|---|---|---|---|
| Perforated carbon on gold grids | 10-50 | Quantifoil | Au 300 R1.2/1.3 |
| Au wire, 0.2 mm, 99.99 % | 21 cm | EMS | 73100 |
| Tungsten wire, 1 mm, 99.95% | 10 cm | Goodfellow | LS339822 |
| Clean glass slide | 1 | | |
| Cover slip | 1 | | |
| Clean wipes (Tekwipe or similar) | | | |

## Equipment

| Description |
|---|
| Tweezers, Dumont N5 or 5 |
| Wrist grounding strap |
| Dissecting microscope |
| Thermal evaporator with $10^{-7}$ torr base pressure, gold source, crystal thickness monitor and shutter |
| Plasma chamber with ultrapure (N6.0 grade) argon and oxygen |

## Detailed procedure

### Grid preparation

Wear grounding strap to prevent static damage to grids during handling.

1. Inspect grids in dissecting microscope and discard any with defects. All grids should be flat, continuous and without dust or lint.

2. If grids have residual plastic from lithographic processing, they can be cleaned by dunking them sequentially in chloroform, acetone and isopropanol (semiconductor grade) and then dried in air.

3. Place grids, carbon side up, on clean glass slide (Figure 1). The grids should be placed as close together as possible without overlap.

4. (Optional) Place a glass cover slip beside the grids. This is used as a shadow mask and for testing if necessary.

#### Preparation of evaporation source

Handle all items used in vacuum with gloves to prevent fingerprints and contamination.
Use a thermal point source made from a 10 cm length of 1 mm diameter tungsten wire with a V-shaped bend in the center. Bake the filament at $> 800$ °C (glowing orange) for $>10$ min in vacuum to remove any surface contaminants before adding the gold wire. This is only necessary before the first use or after a long storage period.

5. Wrap gold wire snugly around the tungsten rod at the V bend (Figure 2).

6. Mount filament in the clamping mechanism across the current source.

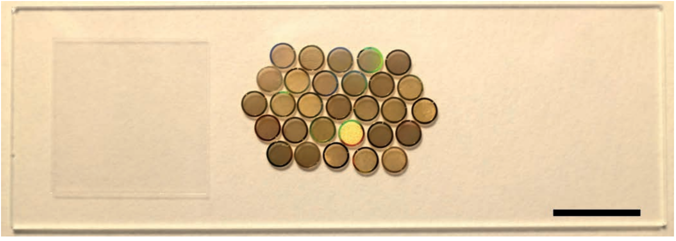

Figure 1: Image of grids on glass slide. Scale bar is 10 mm.

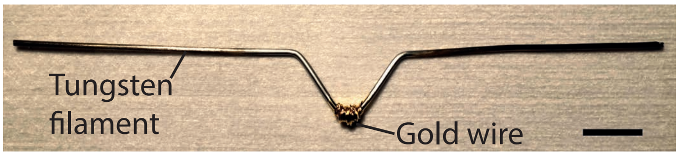


Figure 2: Image of tungsten wire filament prepared with gold wire. Scale bar is 10 mm.

**Evaporation**

We use an Edwards 306 Turbo evaporator equipped with a turbo-molecular pump, a liquid nitrogen cold trap, and an Inficon crystal thickness monitor with water cooling.

7. Start with evaporator in standby: gate valve closed, chamber roughed out and sealed. Fill cold trap with liquid nitrogen to prevent any backstreaming from the pump (cold trap hold time is $\simeq$ 2.5 hrs).

8. Vent chamber with dry nitrogen and remove bell jar.

9. Mount source at a distance $d$ from gold to sample equal to the distance to the crystal thickness monitor ($\simeq$ 130 mm). The source should be horizontally level and centered on the stage (Figure 3).

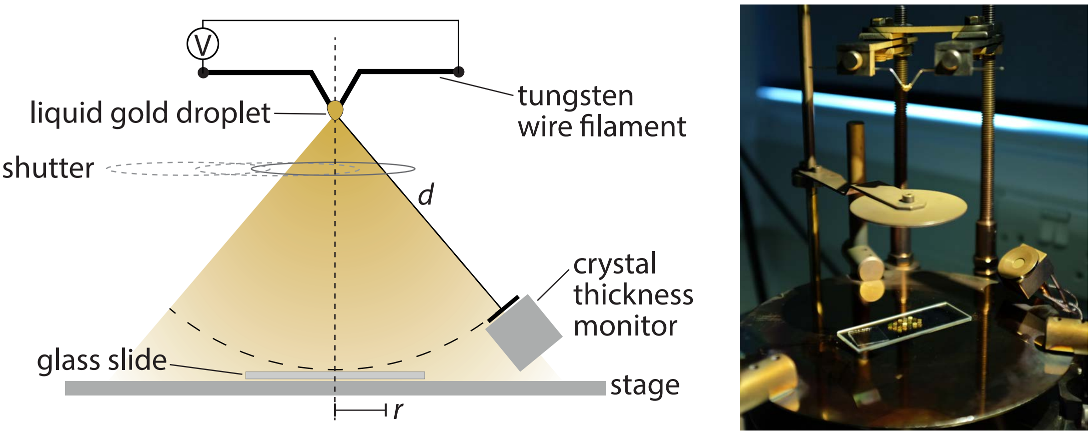


Figure 3: Diagram and photo of evaporation setup.

10. Place glass slide on the stage with grids centered on a vertical axis through the gold source. The thickness $h$ of the film will decrease from the nominal value $h_0$ at the center with increasing $r$ according to

$$\frac{h}{h_0} = \sqrt{1 - \frac{r^2}{d^2}} \tag{1}$$

so this should be considered when determining how many grids to place at a time for a given source to sample distance $d$.

11. Check that the shutter can block deposition on the grids while not obscuring the crystal thickness monitor. Take care that the penumbra of the shutter does not fall on either the sensor or the glass slide.

12. Check that the gasket on the bell jar is clean; place bell jar over the evaporator setup and evacuate chamber.

13. Turn on crystal thickness monitor and set for gold (density = 19.3 g/mL, Z factor = 0.381 tooling factor = 100; program 2 on our system). Turn on water cooling for crystal.

14. Turn on LT power and heat source to $\simeq$ 1 amp for 5 min during pump down to burn off water and other contaminants without melting the gold.

15. Turn off current and continue to pump chamber until pressure is $\simeq$ 1x$10^{-7}$ torr, topping up liquid nitrogen if necessary. Do not let the liquid nitrogen cold trap warm up while the gate valve is open (i.e. no overnight or unattended pump down).

16. Slowly heat up filament over 3—5 min until it is glowing.

17. Once the wire is melted, heat up a bit more (probably around 1.6 Amps) and monitor gold deposition rate using the crystal thickness monitor. Adjust current until the deposition rate is 1.0 $\pm$ 0.1 Å/sec. Pressure will increase to $\simeq$ 8x$10^{-6}$ – 1x$10^{-5}$ torr during evaporation.

18. To begin depositing gold onto the grids, open the shutter. Zero the crystal thickness monitor as the shutter opens.

19. Close shutter after 450 Å gold has been deposited. Turn down current and switch off LT.

20. Let cool $>$ 30 min.

21. Vent chamber with dry nitrogen and remove grids. Gold on the inside of the bell jar is removed easily with a Tekwipe and compressed air.

**Plasma removal of carbon**

After evaporation of gold, the carbon is removed by low-energy plasma treatment. We use a Fischione model 1070 with argon and oxygen.

22. Load grids into plasma chamber. We use a custom suspension holder but the grids can also be placed on a clean glass slide, carbon side up on the shelf in the chamber.

23. Evacuate to $\ll 10^{-4}$ torr.

24. Admit high purity argon and oxygen (BOC 99.9999%) in a ratio of 9:1 to a pressure of 21 mtorr.

25. Apply radio frequency plasma with 38 W of forward power and $\leqslant$ 2 W of reverse power for $\geqslant$ 6 min.

26. Store grids in a low-humidity, dust-free container until used.

**Notes**

- When adapting this process to other systems, the most important parameters to reproduce are pressure, sample temperature during evaporation and deposition rate.
- Glass slides, as purchased, can be cleaned by sonicating in 2% Micro90 or similar detergent and rinsing with ultrapure, 18 M$\Omega$ deionized water.
- Calibrate crystal thickness monitor using AFM or other independent thickness measurement.
- Source to sample distance during evaporation is important as it can affect the heating of the substrate during the evaporation and the uniformity of the foil thickness across the batch of grids.
- The carbon etch rate of plasma treatment should be calibrated to ensure complete removal.
- Higher energy plasmas may damage the gold by sputtering.
