# Supplementary material for: Ultrastable gold substrates: Properties of a support for high-resolution electron cryomicroscopy of biological specimens
Source: J Struct Biol. 2016 Jan;193(1):33–44. doi: 10.1016/j.jsb.2015.11.006 (PMC4711342; doi:10.1016/j.jsb.2015.11.006)
Supplement: Supplementary file 2 — Experimental protocol for the float transfer of thin films of carbon to ultrastable supports. [file mmc2.pdf]

Carbon Float procedure

Reference:
Ultrastable gold substrates: Properties of a support for high-resolution electron cryomicroscopy of biological specimens
Christopher J. Russo & Lori A. Passmore

Summary

This procedure describes how to float thin amorphous carbon films onto support grids for electron microscopy, including ultrastable gold supports [1].

Materials

Description	Amount
Support grids, e.g. Quantifoil, UltrAuFoil	1–50
Amorphous carbon on mica	1 sheet, $\simeq$ 2.5 x 2.5 cm
Whatman No. 1 filter paper, diameter 70 mm	5
DI H_2O filtered, 18 MΩ	0.5 L

Equipment

Tweezers, Dumont N5 or 5, clean by solvent rinse (plasma clean if necessary)
Wrist grounding strap
Dissecting microscope
Glass crystallisation dish (Pyrex), diameter 90 mm, height 50 mm
600 mL beaker to collect waste water
Stainless steel ring with polished and bevelled edges, 2 mm thick, height 10 mm, diameter 50 mm
Stainless steel mesh, diameter 65 mm, made with 0.7 mm wire with 3 mm mesh
Tubing for siphon, length 0.6 m, diameter 3.2 mm (outer), 1.6 mm (inner)
Disposable syringe

Detailed procedure

Grid preparation

Wear grounding strap to prevent static damage to grids during handling. Don't wear a glove in the hand that holds the tweezers, or ground the tweezers directly.

1. Inspect grids in dissecting microscope and discard any with defects. All grids should be flat, continuous and without dust or lint.

2. If grids have residual plastic from lithographic processing, they can be cleaned by dunking them sequentially in chloroform, acetone and isopropanol (semiconductor grade) and then dried in air.

Preparation of float chamber

Handle all items with gloves to prevent fingerprints and contamination.

3. Place stainless steel mesh, filter paper and stainless steel ring in the crystallization dish as depicted in Figure 1.

4. Fill dish with DI water (18 MΩ, filtered, UV treated). Overfill it to break the meniscus at the surface and remove any contaminating surface layers on the water. Pour off excess water so the level is just below the rim of the glass dish.

5. Using tweezers, carefully place grids, foil side up, onto the centre of the filter paper. They should enter the water at an angle.

6. Set up the siphon: attach tubing to dish with normally closed forceps or a spring clip. Start flow of syphon with syringe and clamp off flow while floating the carbon (next step).

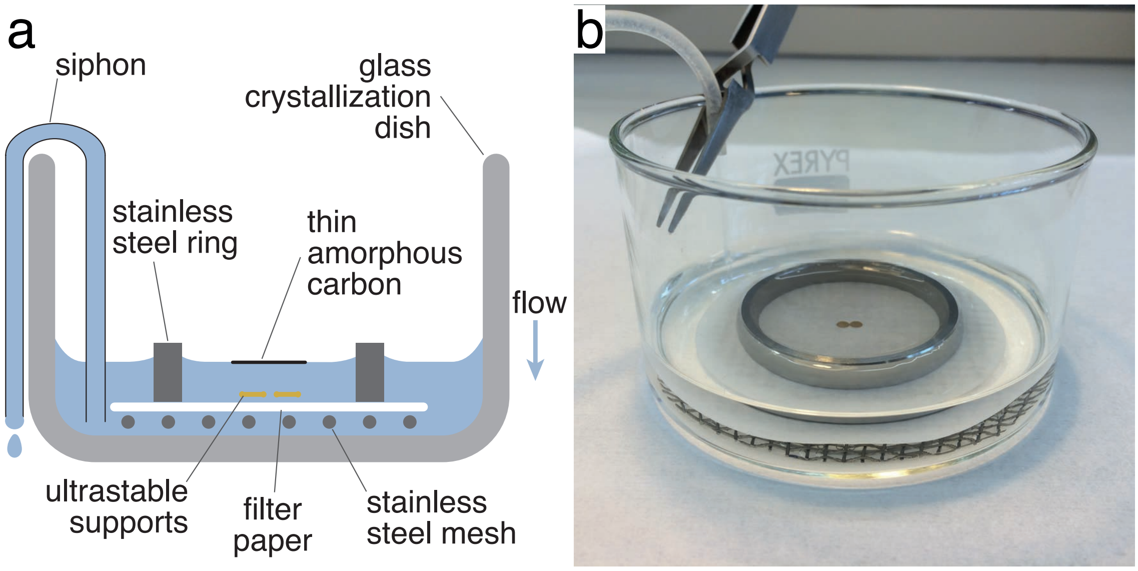

Figure 1: Apparatus for depositing thin films of amorphous carbon on gold foils. Panel **a** shows a cross-sectional diagram of the float chamber. As the water level is lowered, the thin film of amorphous carbon is deposited onto the supports. Panel **b** shows a photo of the apparatus in use.

Deposition of carbon

7. Float carbon off mica by slowly lowering the mica (carbon-side up) into the water at an angle. A light can be placed to shine off the surface of the water at a glancing angle, allowing you to see the carbon better.

8. Use siphon to slowly lower the water level.

9. Monitor the position of the carbon with respect to the grids and ensure it stays centred by gently nudging with clean tweezers.

10. Once the carbon has been deposited on the grids and the water level is below the filter paper, carefully lift off the stainless steel ring. Remove the filter paper and mesh together. Place on a dry filter paper and cover with a clean glass petri dish or beaker, tilted slightly to leave room for evaporation. Allow to dry.

11. When dry, store in a clean *glass* petri dish until ready to use.

Notes

We recommend using only glass (no plastic) petri dishes and beakers for all grid processing and storage.

References

[1] C. J. Russo and L. A. Passmore. Ultrastable gold substrates for electron cryomicroscopy. *Science*, 346(6215):1377–1380, Dec. 2014.
