# Supplementary figures and images for: Ultrastable gold substrates: Properties of a support for high-resolution electron cryomicroscopy of biological specimens
Source: J Struct Biol. 2016 Jan;193(1):33–44. doi: 10.1016/j.jsb.2015.11.006 (PMC4711342; doi:10.1016/j.jsb.2015.11.006)

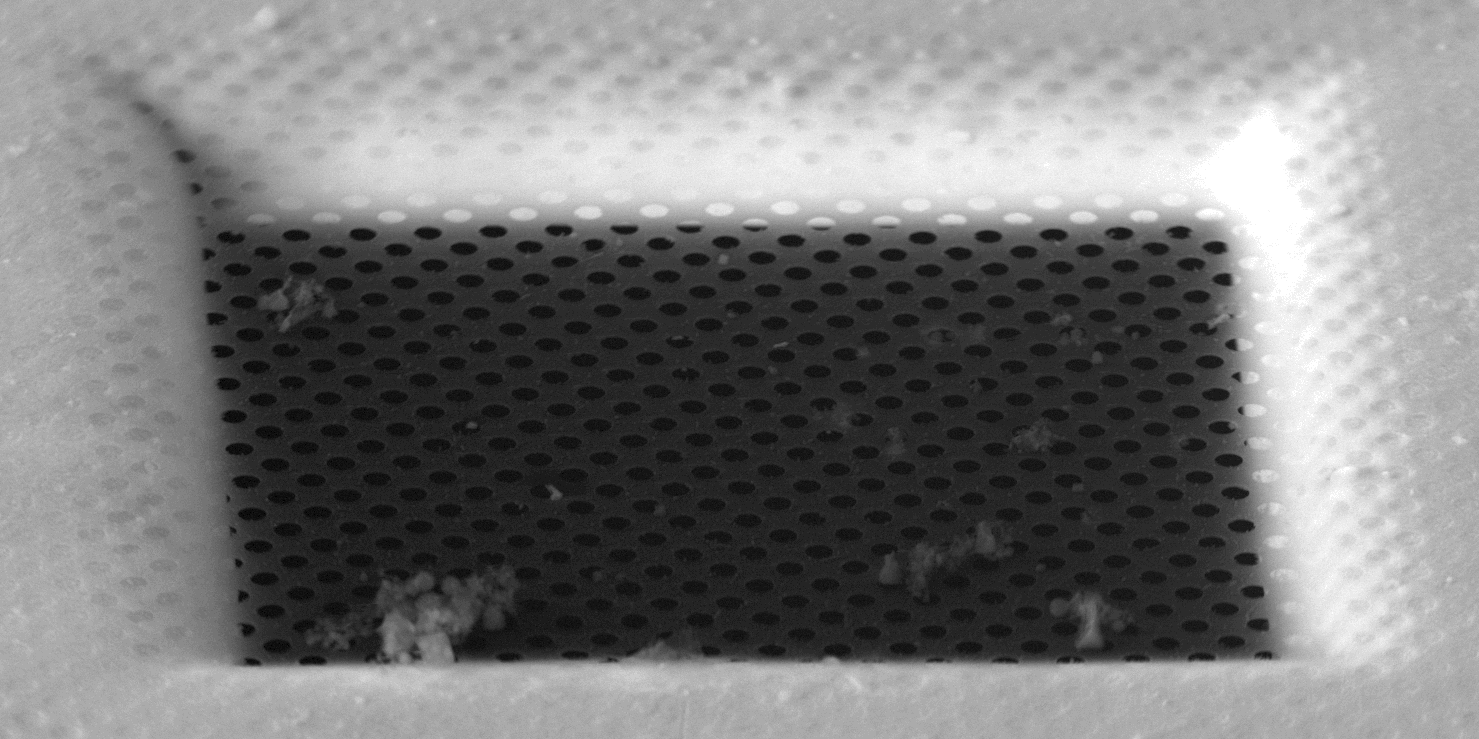

Supplement: Supplementary video 1 — Animated before and after scanning electron cryomicrographs of a suspended, perforated amorphous carbon foil irradiated with a high dose in a local region under cryogenic conditions. Field of view is 80 μm wide and tilt of specimen is 59° along the horizontal axis. [file mmc3.gif]

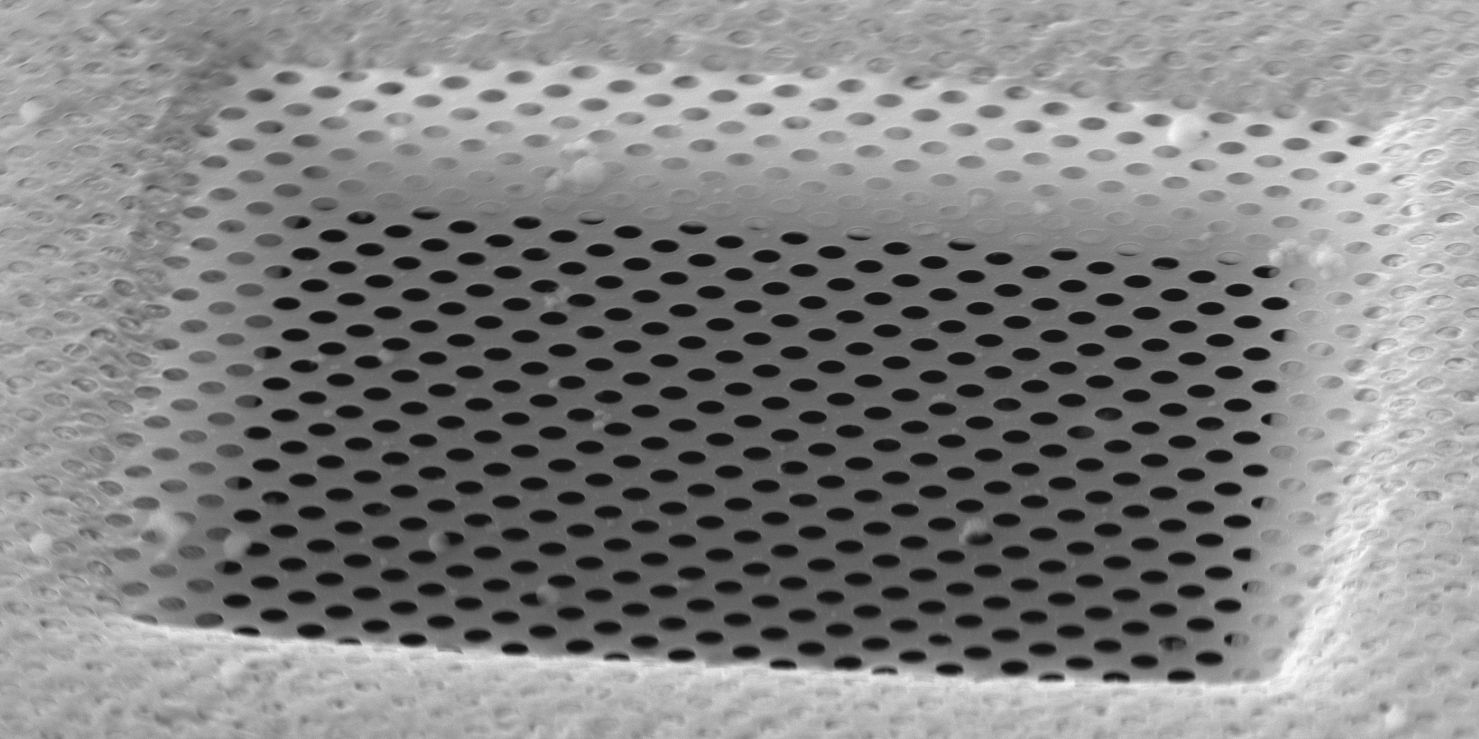

Supplement: Supplementary video 2 — Animated before and after scanning electron cryomicrographs of a suspended, perforated gold foil irradiated with a high dose in a local region under cryogenic conditions. Field of view is 80 μm wide and tilt of specimen is 59° along the horizontal axis. [file mmc4.gif]

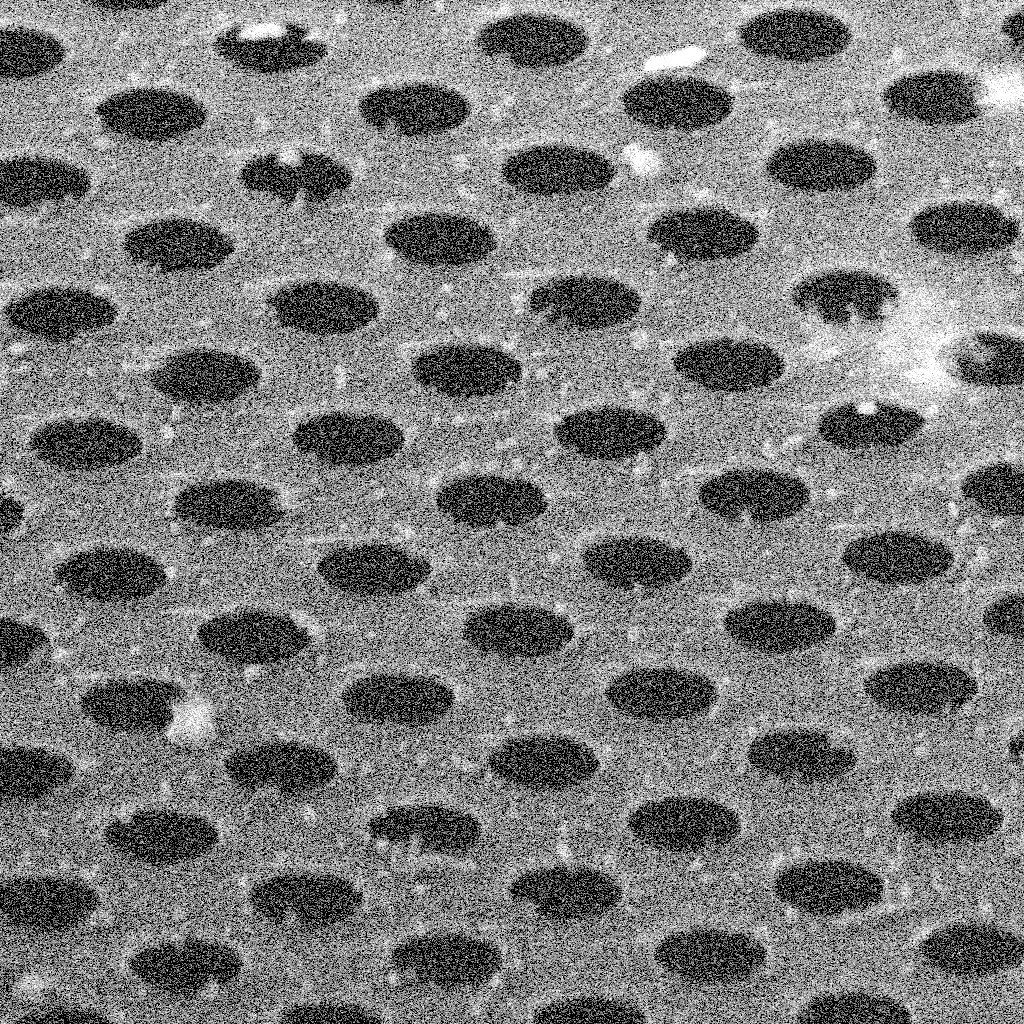

Supplement: Supplementary video 3 — Enlarged view of the local region irradiated in Video 3, showing the bending and movement of the foil. Irradiated patch is 5 μm wide. [file mmc5.gif]

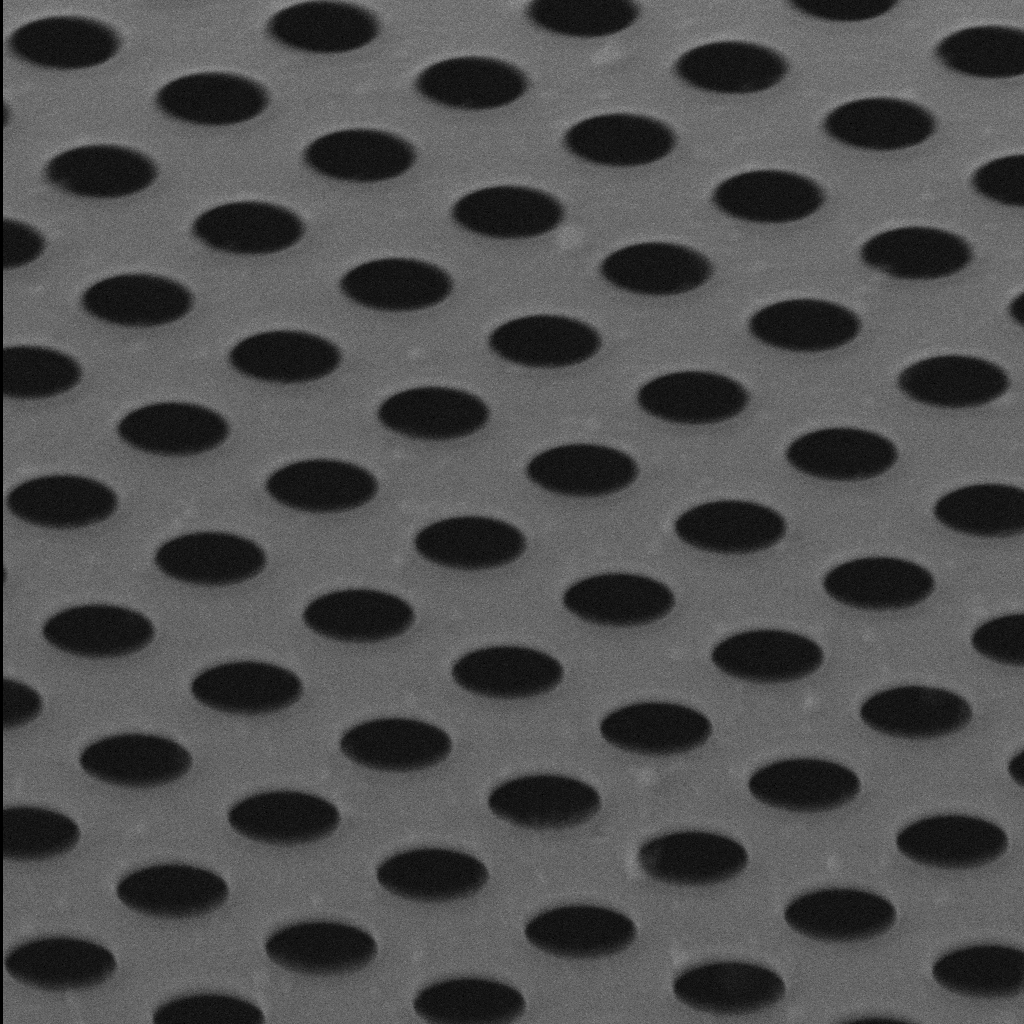

Supplement: Supplementary video 4 — Enlarged view of the local region irradiated in Video 4, showing the bending and movement of the foil. Irradiated patch is 5 μm wide. [file mmc6.gif]
